# Comparison of mid-term efficacy of spastic flatfoot in ambulant children with cerebral palsy by 2 different methods: Erratum

**DOI:** 10.1097/MD.0000000000010690

**Published:** 2018-05-04

In the article, “Comparison of mid-term efficacy of spastic flatfoot in ambulant children with cerebral palsy by 2 different methods”,^[1]^ which appears in Volume 96, Issue 22 of *Medicine*, Dr. Jie Wen should be affiliated with “1.Department of Pediatric Orthopedics, the Children's Hospital of Fudan University, Shanghai, China” and “2.Department of Pediatric Orthopedics, Hunan Provincial People's Hospital, Changsha, Hunan, China.”
